# The University and Hospital Bulletin

**Published:** 1905-01

**Authors:** 


### The University and Hospital Bulletin

DEVOTED TO THE INTERESTS OF THE UNIVERSITY OF BUFFALO AND THE HOSPITALS.

EDITED BY

NELSON W. WILSON, M. D.

*Assistants:*

E. R. McGuire, M. D., Burton T. Simpson, M. D.

Under the auspices of the Faculty of the Medical Department of the University

M. D. Mann, A. M., M. D., Dean,
Charles G. Stockton, M. D.,
John Parmenter, M. D.,
Eli H. Long, M. D.,
Roswell Park, A. M., M. D., LL. D.,
Charles Cary, M. D.,
Herbert M. Hill, A. M., Ph. D.,
Herbert U. Williams, M. D.

AND

*Buffalo General Hospital* Henry Reed Hopkins, M. D.
*Hospital of the Sisters of Charity* Eugene A. Smith, M. D.
*German Hospital* Herman E. Hayd, M. D.

The Christmas holidays began on the 17th of December and will continue until January 3, 1905, when the work of the medical department will be resumed.

---

The annual theatre party of the students of the University was held on December 5, Shea's being the theatre chosen. The playhouse was decorated with the University blue and white. The vaudeville bill was enlivened by the actors at apropos moments during their acts by references to some of the students. The four departments of medicine, law, pharmacy, and dentistry were well represented, the classes of each department occupying sections of the theatre. The class songs were brighter this year than they have ever been and the class yells greeted popular teachers as well as brilliant bits of the program.

---

The pathological department of the University received Thanksgiving time the organs of a turkey which had been purchased in open market and which by reason of their peculiar appearance excited suspicion of disease. A preliminary examination has been made and the liver and lymph nodes have been found to be tubercular. Further study is being made and when completed a full report will be published.

---

A rather unusual fracture of the tibia was received at the General Hospital during the month of November. A traveling man alighting from a slowly moving train at Columbus, O., fell on a heap of stones; the train was stopped and he was taken aboard and brought to Buffalo, a physician, en route, putting on an emergency splint of book covers and a cotton bandage. When the patient was received at the hospital in Buffalo, sixteen hours after the accident, the leg was enormously swollen and covered with blebs. The injury was found to be a multiple fracture of the head of the tibia. No immediate effort was made to reduce the fractures, the first treatment consisting of cleaning off the surface and immobilising the limb. Ice bags were applied. A radiograph showed the outer head of the tibia had been broken off; a fracture extended diagonally across the bone from the inner head to the outer surface of the shaft, an inch below the first fracture, and an almost perpendicular fracture from the middle of the second fracture to the inner surface of the shaft. This last fracture was broken across transversely. After three weeks in plaster there was fair union of the fragments which had been replaced as nearly as possible under anesthesia. In many such cases where there are multiple fractures of the head of the tibia, involving the joint, there is non-union and amputation is necessary. Invariably the result when the leg is saved is a stiff joint.

---

At the surgical clinic of the General Hospital a woman of 40 years was received whose symptoms were referable to the stomach and there were indications which made the suspicion of carcinoma tenable. An exploratory laparatomy showed the stomach to be in normal condition. A further examination revealed a very large gallbladder which contained stones. The bladder was removed and on being opened was found to be filled with bile in which were a large number of stones varying from very coarse sand to pea size. They were counted and numbered over 1,600.

---

Repair of fractured pelvis was done at the surgical clinic of the General Hospital, the patient being a man of 60, who had sustained the injury sometime prior to his admission. He could walk fairly well, but with considerable pain, which when he sat down was intensified, and there were few moments when he did not have some pain more or less severe in character. Examination showed a separation of the symphysis and a fracture of the ascending ramus of the ischium. The latter was fairly solid with fibrous union and consequently was not disturbed. The upper fracture was wired with very heavy silver wire and the case made a good recovery.

A GIRL of 13 came to the General Hospital surgical clinic with ankylosis of the jaw which so far as could be learned was a sequel of one of the exanthema. The child could not open her mouth more than half an inch and even this was attended with great difficulty. Resection was done just a little anterior to the angle of the jaw, on both sides, and was completely successful, the child on recovery having excellent control of the jaw movements.

---

THE complications which may and frequently do arise in strangulated hernias was forcibly shown in a case operated at the surgical clinic of the General Hospital within the past few weeks. The patient was a woman 40 years of age who was received with a strangulated umbilical hernia which had resisted all efforts of her attending physician to reduce. She was hurriedly prepared for operation and on opening the abdomen the hernia was found to be in a condition of approaching gangrene. There was an abundance of pus in the abdominal cavity and an examination of the appendical region disclosed a gangrenous appendix which was removed. The patient's condition was precarious and in view of the extensive suppurative condition which existed recovery was considered doubtful. She, however, rallied from the operation rapidly and is now believed to be convalescent.

---

A MOST interesting case of abdominal tumor was received at the surgical clinic of the General Hospital, in which the diagnosis was made and operation done for cystic tumor of the liver. The patient was a German woman who had been in this country for eighteen years. There was a large tumor to the right of the middle line of the abdomen in its upper quadrant. The history of the case was rather obscure, but mention was made of blood stained feces. There was a sense of pressure in the region of the tumor, this pressure apparently being directed upward, causing some difficulty in respiration. The question to be determined in diagnosis was whether the tumor was an enlarged gallbladder, a pancreatic cyst, or a cystic tumor of the liver. The latter diagnosis was made and the abdomen opened, revealing a large tumor of the liver, cystic in type, which was opened and drained. The patient died within a short time after the operation and an autopsy was performed. The pathologic findings were interesting, showing the tumor to have been a hydatid cyst of the liver. The peritoneum was clean and there were no signs of infection elsewhere. The pathologic report of the case will be made at a later date. There was no trace of the presence of hooklets in the urine prior to operation.

UNIVERSITY ATHLETICS.

DR. BURTON T. SIMPSON read a paper before the faculties of the departments of medicine, law, pharmacy and dentistry at the university building on Saturday afternoon, December 17, 1904, on athletics. The paper was entitled "Consideration of the advisability of athletics in professional colleges with special reference to the University of Buffalo." Over a hundred invitations had been sent out for the meeting and nearly fifty were present when Dr. Simpson began his paper, which was most interesting and showed a large amount of work in the preparation of statistics as well as in the presentation of facts and argument. It was fully discussed by members of the several faculties and will be published in a future edition of the JOURNAL, together with an abstract of the discussion.

